# In Vitro Screening Method for Characterization of Macrophage Activation Responses

**DOI:** 10.3390/mps5050068

**Published:** 2022-08-30

**Authors:** Brandon W. Lewis, Sonika Patial, Yogesh Saini

**Affiliations:** Comparative Biomedical Sciences, School of Veterinary Medicine, Louisiana State University, Baton Rouge, LA 70803, USA

**Keywords:** macrophage, BMDM, macrophage activation, M1, M2, alternatively-activated macrophages (AAM), classically-activated macrophages (CAM)

## Abstract

Macrophage activation refers to the enhanced functionality of macrophages in response to endogenous or exogenous stimuli. Due to the existence of limitless stimuli and a multitude of receptors on macrophage surfaces, the nature of activation (or acquired functioning) can be specific to the encountering stimulus. This article describes a macrophage-activation screening platform in a 96-well format. The methodology involves the generation of bone marrow-derived macrophages, their activation into two extreme activation states, and screening of activated macrophages for expression of bonafide protein biomarkers. A high-throughput and stringent assay to determine macrophage activation markers developed in this article can be adapted for biomarker determination in pathological conditions and toxicant/drug safety screening.

## 1. Introduction

Local alteration in homeostatic tissue environment is sensed by resident macrophages and other innate immune cells [1,2,3,4,5]. Based on the nature and severity of perturbations, tissue macrophages boost their functional armor via increased expression of genes involved in pathogen killing, wound healing, cytokine secretion, and phagocytic clearance [6]. Despite clear-cut categorization of activated macrophage populations into M1 (classically activated) and M2 (alternatively activated), the activation markers identified in macrophages obtained from different tissue compartments and disease models reflect a great degree of heterogeneity in macrophage activation responses [2,7].

M1 macrophage activation is induced by Interferon (IFN)-γ and lipopolysaccharide (LPS) [8]. These macrophages express pro-inflammatory cytokines including Interleukin (IL)-6, IL-1β, and TNFα, and inducible nitric oxide synthase (iNOS) [9,10]. M2 macrophages are divided into three subcategories, i.e., M2a, M2b, or M2c, based on the expression of surface markers, production of specific cytokines, and their acquired functions [11]. M2a activation is induced by IL-4 and/or IL-13 and they express markers including Arg1 and Fizz1 [8,12]. M2b or regulatory macrophages are induced by LPS and immune complexes and secrete IL-10. M2c macrophages are induced by IL-10 and secrete profibrotic, i.e., TGFβ and anti-inflammatory, i.e., IL-10, mediators [13,14].

Identification of macrophage activation patterns can be performed in purified macrophages from healthy and diseased tissues using gene expression profiling [15,16], western blotting, immuno-cytochemical staining, and flow cytometry [17,18]. Although employed routinely, these approaches generally pose challenges, including the selection of effective reagents (antibodies), tissue/cell processing, and lack of positive controls. In our attempt to develop a robust, reliable, and consistent assay, after screening various antibody specificities using protein electrophoresis/western assays, we employed an in-cell western technology to develop a macrophage activation screening platform. In-cell western offers many advantages compared to traditional assays such as immuno-cytochemistry and western blotting. It provides higher sensitivity and more meaningful protein expression analyses in whole cells. This approach allows for enhanced quantitative analyses in a high throughput manner in plated whole cells. Importantly, this approach allows reduction in the number of animals used in research. Although developed using BMDM cell line, the assay can be adapted to various primary macrophage (Bronchoalveolar/peritoneal macrophages) and immortalized macrophage cell lines.

## 2. Materials

Table 1 summarizes the reagents and equipment utilized in this study.

## 3. Procedure

### 3.1. Generation of Macrophage-Colony Stimulating Factor (M-CSF) Containing L929 Medium

**Figure 1:**
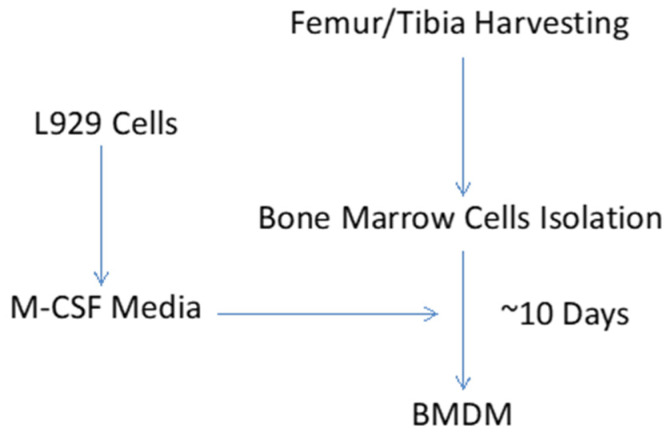
BMDM Generation.

An overview of experimental procedure describing generation of BMDMs is shown in Figure 1.

(1) Plate 10^6^ L929 cells (M-CSF producing cell line, CCL-1, was purchased from ATCC) in 20 mL of DMEM (Gibco, # 11995-065) supplemented with 10% heat inactivated fetal bovine serum (FBS), and 1% penicillin/streptomycin (Lonza, #17-602E) in a T75 cell culture flask.
(2) Grow cells for one week (or until the flask is ~90% confluent) at 37°C, 5% CO_2_. It is important to keep the seeding density and harvesting schedule consistent to avoid variation in L929 concentration is the L929 medium.
(3) Collect the medium and spin at 400× *g* for 5 min to remove any floating cells and cellular debris.
(4) Filter the supernatant through a 0.22 μm filter. Store the filtered L-929 medium in 150 mL aliquots at −20 °C.
(5) L929-conditioned macrophage media is then prepared by mixing L-929 medium (30%) and RPMI 1640 (Caisson Labs, #RPL09-500 mL) complete medium (70%).

### 3.2. Collection of Bone Marrow Cells

(1) Euthanize six-week-old male mice on C57BL/6J background by CO_2_ inhalation and cervical dislocation. Disinfect euthanized mice with 70% ethanol and betadine (this step can be performed on bench-top).
(2) Using sterile surgical instruments, isolate femur and tibial bones, and transfer to 100 mm cell culture dish containing 5 mL of RPMI 1640 complete media (10% FBS, and 1% pen/strep).
(3) Transfer cell culture dish containing bones to cell culture hood for further processing. Using new set of surgical instruments, cut bones from both ends to expose the marrow cavity. Flush the bone marrow into new cell culture dish with 10 mL syringe (containing RPMI 1640 complete media) fitted with 22G needle.
(4) Dissociate bone marrow with repeated passing through 25G needle. Strain the cell suspension through 70 μm cell strainer in to 50 mL sterilized conical tube.
(5) Spin the cell suspension at 400× *g* for 5 min. Resuspend the cell pellet in 20 mL of L929-conditioned macrophage medium and plate in T25 cell culture flask for overnight incubation. Next morning (~12 h wait), replate non-adherent cells from T25 flask into 100 mm petri dishes (20 dishes per mouse) for differentiation of bone marrow cells to macrophages.
(6) Add 3 mL fresh L929-conditioned macrophage medium every day. Switch L929-conditioned macrophage medium at 3-day intervals (7 mL per dish). Collect adherent cells (mature macrophages) on 10th day of culture and proceed to in vitro macrophage activation step.

### 3.3. BMDM Macrophage Activation

**Figure 2:**
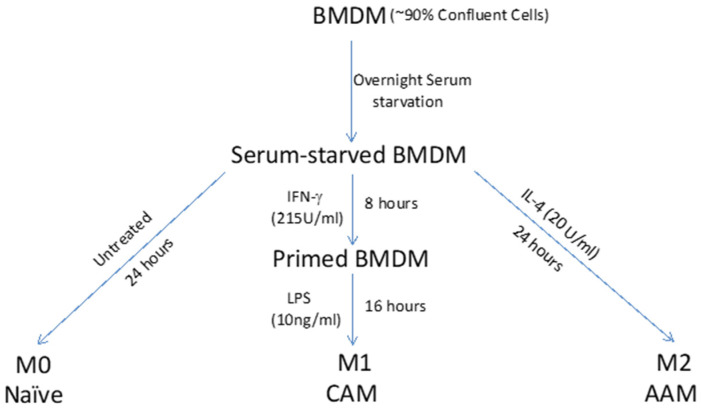
Macrophage Activation.

A step-by-step protocol for generation of activated macrophages is shown in Figure 2.

(1) Seed BMDMs in designated wells (seeding density: 40,000 per well in 200 μL volume) of 96-well cell culture plate. Incubate the plate at 37 °C for 1 h.
(2) To prevent the likely influence of serum constituents on the macrophage responsiveness at baseline, replace the media with serum-free RPMI 1640 complete medium, and incubate the plate for overnight serum starvation. Of note, in case of possible loss of viability of cells due to serum-free environment, RPMI 1640 complete medium with 1–2% serum could be used.
(3) Next day, label wells for M1, M2, and M0 (non-activated). Replace media (24 wells/treatment) with fresh serum-free RPMI 1640 complete medium containing IFN-γ (215 U/mL) + LPS (10 ng/mL; added 8 h after the start of IFN-γ treatment) for M1 activation or IL-4 (20 U/mL) for M2 activation. Wells assigned to M0 group will receive fresh serum- free RPMI1640 complete medium with no additives [8].

### 3.4. Screening for Macrophage Activation Markers

**Figure 3:**
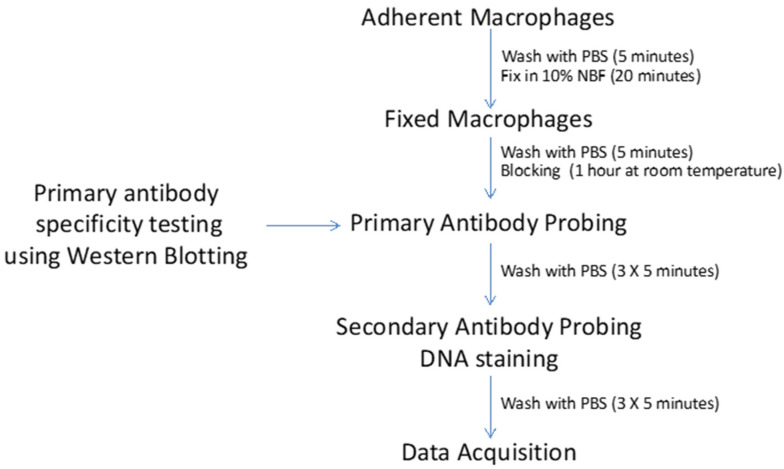
Macrophage Activation Marker Analyses.

A detailed overview of macrophage activation screening is shown in Figure 3.

(1) Wash once with 1× PBS buffer and fix with 100 μL of 10% neutral buffered formalin (Fisher Scientific) for 20 min, at room temperature. Use of multichannel pipette is highly recommended. To avoid dislodgment of cells, it is important to add buffers/solutions alongside the walls.
(2) Centrifuge the 96 well plate at 500× *g* for 5 min. Aspirate the formalin fixative and wash cells once with 1× PBS.
(3) Wash cells three times (5 min for each wash) with 0.1% Triton X-100 PBS (1× PBSTr) to permeabilize cells.
(4) Wash cells once with 1× PBS. Add 200 µL of LI-COR Odyssey blocking buffer per well to block cells. Incubate on a rocker for 1 h at room temperature.
(5) Add 50 μL of primary antibody solution (prepared in Odyssey Blocking Buffer) to each well and incubate overnight on rocker in a cold room.
(6) Wash the plate three times with 0.1% Tween-20 PBS (1× PBSTw) for 5 min at room temperature.
(7) Incubate with 50 μL of goat anti rabbit IRDYE 800CW secondary antibody solution (1:1000) for 1 h at room temperature. At this step, include DNA labeling dye (1:10,000 DRAQ5 stain) for normalizing cell numbers in each well.
(8) Wash with 1× PBSTw for 5 min, three times.
(9) Wash with 1× PBS for 5 min. Discard wash solution and dry the plate on paper towels.
(10) Scan the plate in both the 700 nm and 800 nm detection channels using Odyssey Imager (Odyssey CLx Infrared Imaging System, Lincoln, NE). Normalize the primary antibody signal by dividing value obtained on 800 nm channel with values obtained on 700 nm channel for each well.

## 4. Results

**Figure 4:**
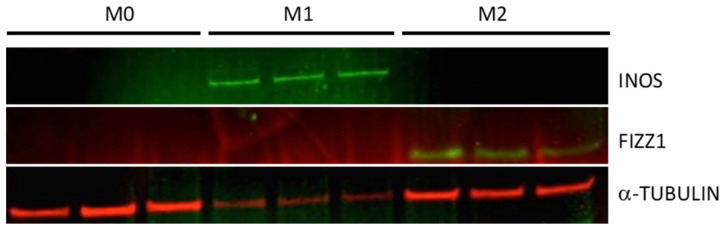
Western blot analyses on Naïve (M0), M1-activated, and M2-activated BMDMs.

First, we tested the specificity of antibodies for bonafide M1 and M2 markers by performing western blots (Figure 4). The protein expression of INOS, a bonafide marker to test M1 activation, was enhanced in classically activated macrophages, whereas the protein expression of FIZZ1, a bonafide marker to test M2 activation, was enhanced in alternatively activated macrophages.

**Figure 5:**
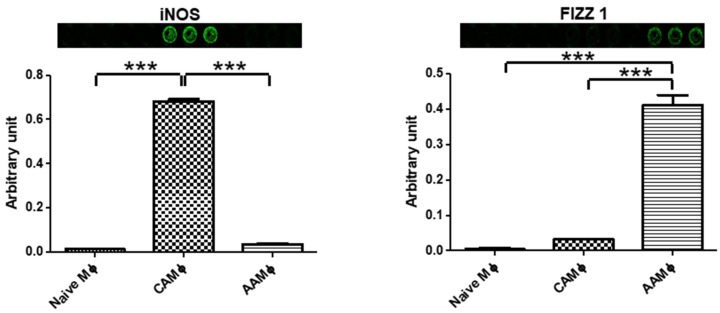
In Cell Western analyses for activation-specific markers in Naïve (M0), M1-activated (CAM), and M2-activated (AAM) BMDMs. N = 3, ANOVA, *** *p* < 0.001. Error bars represent standard error of the mean (SEM).

INOS (M1 marker) and FIZZ1 (M2 marker) expression status was interrogated using in cell western approach (Figure 5). Similar to the results obtained with western blots (Figure 4), INOS was enhanced in classically activated macrophages while FIZZ1 was enhanced in alternatively activated macrophages in in cell western assays.

**Figure 6:**
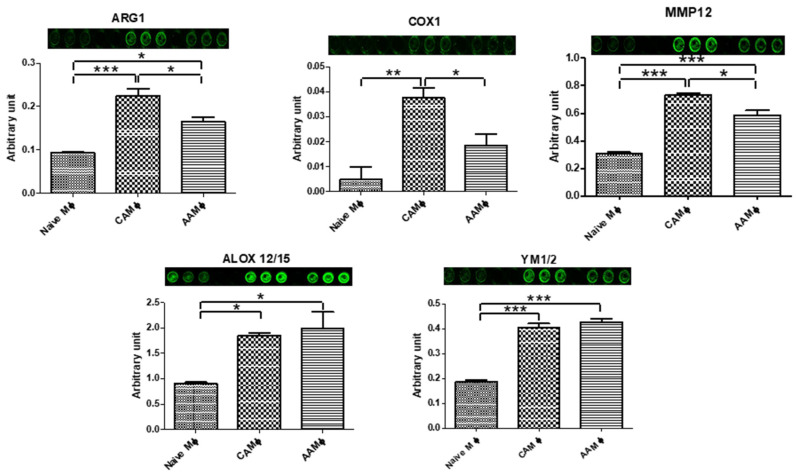
In Cell Western analyses for activation-specific markers (ARG1, COX1, MMP12, ALOX12/15, and YM1/2) in Naïve (M0), M1-activated (CAM), and M2-activated (AAM) BMDMs. N = 3, ANOVA, * *p* < 0.05, ** *p* < 0.01, *** *p* < 0.001. Error bars represent standard error of the mean (SEM).

Antibody specificity for other activation markers (ARG1, COX1, MMP12, ALOX12/15, and YM1/2) was confirmed using western blot (data not shown). Finally, activated macrophages were interrogated for the expression levels of selective activation markers (Figure 6).

## 5. Discussion

In this article, we report a well-standardized and highly adaptive in vitro method to assess macrophage activation patterns based on the expression of activation-specific protein markers. Although the current study is focused on BMDM activation responses to M1 and M2 treatments, the method can be easily modified for any other macrophages, including other primary macrophages and macrophage cell-lines. In addition, the method offers multiple advantages in research. First, the 96-well format allows simultaneous screening of large number of test samples. Additionally, few wells of 96-well plates can be seeded with known activated and naïve macrophages that can serve as positive and negative controls during analyses of unknown samples. Second, the method can be adapted for high throughput screening of drugs/toxicants for safety assessment. Third, the method can be adapted for phenotypic investigation of macrophage populations from various murine models of diseases.

One caveat of this method is the inability to simultaneous label cells for more than one marker. This limitation is commonly encountered in most of the current techniques except flow cytometry. However, the in vitro method described in this study presents a robust tool as a first line of investigation on macrophage responses in toxicant/drug screening as well as disease investigation. While this protocol was established using murine BMDMs, it can be used for other types of macrophages, including human monocytes derived from peripheral blood mononuclear cells and macrophages derived from other species. However, differentiation of mononuclear cells into macrophages would require species-specific protocols.

Inducible nitric oxide synthase (INOS) is the signature M1-activation marker that is characteristic of classical activation response [19]. As expected, INOS expression was only observed in classically activated macrophages. For M2 activation, we specifically focused on M2a subset that is induced by IL-4. Our method can be adapted to macrophages stimulated with TFGβ and IL-10 (M2c). For M2-specific signatures, we used found in inflammatory zone 1 (FIZZ1) and chitinase 3 like 4 (CHI3L/4) markers [12,20]. FIZZ1, a well-standardized M2 marker, was only seen in M2 macrophages. Although baseline expression of CHI3L3/4 (also known as YM1/2) was observed in M0 as well as M1 macrophages, only M2 macrophages showed significantly upregulated expression. The assessment of the expression levels of these three markers would identify M1 or M2- activated macrophages.

In summary, the assay developed in this article is highly adaptable, high-throughput, and informative and can be utilized in toxicological screening as well as disease phenotyping studies.

## Figures and Tables

**Table 1:** List of reagents.

Reagent	Company/Institute	Cat. Number	Comments
C57BL/6J	Jackson Laboratory	000664	Mouse Strain
L-929 Cell line	ATCC	CCL-1	Mouse connective tissue fibroblast cell line
Dulbecco’s Modified Eagle Medium (DMEM)	Gibco (Life Technologies)	11995-065	With Glucose, L-Glutamine and Sodium Pyruvate
RPMI 1640	Gibco (Life Technologies)	22400-089	With L-Glutamine and HEPES
Fetal Bovine Serum	Atlanta Biologicals	S11550H	Heat Inactivated
Penicillin/Streptomycin (100X)	Sigma-Aldrich	P4333	Working concentration (1X)
Phosphate Buffered Saline (PBS)	Sigma-Aldrich	P5368	0.01 M PBS; pH 7.5
Interleukin 4	EMD Millipore	IL016	Recombinant (Murine)
Interferon Gamma (IFN-γ)	EMD Millipore	IF005	Recombinant (Murine)
Lipopolysaccharide (LPS)	EMD Millipore	LPS25	LPS, *E. Coli* O111:B4
96-well plates	Thermo Scientific	165305	Black with polymer base
Filtration Unit	Genesee	25-227	0.22 μm
Betadine	Purdue Products	67618-151-17	Povidone-iodine, 7.5%
Cell strainer	Corning Life Sciences	352350	70 μm
T25 culture flask	Genesee	25-207	
T75 culture flasks	Genesee	25-209	
100 mm Petri-dishes	Thermo Scientific	130182	
10% Buffered Formalin	Fisher Scientific	SF100-20	pH 7.0
Triton x-100	Fisher Bioreagents	BP151-100	Electrophoresis Grade
Tween-20	Fisher Bioreagents	BP337-100	Electrophoresis Grade
FIZZ1A	ABCAM	AB39626	Rabbit Polyclonal
YM1/2	National Institute of Health	Rabbit Polyclonal	A kind gift from Dr. Shioko Kimura
INOS	ABCAM	AB15326	Rabbit Polyclonal
HIF2a	ABCAM	AB199	Rabbit Polyclonal
COX 1	Cell Signaling	4841	Rabbit Polyclonal
MMP12	ABCAM	AB15326	Rabbit Polyclonal
ARG1	Santa-Cruz Biotechnology	SC-20150	Rabbit Polyclonal
Odyssey Blocking Buffer	LI-COR	927-40000	PBS-based buffer
Odyssey Imager	LI-COR	Odyssey CLx	Infra-red Imager

## Data Availability

The data used and analyzed during the current study are included in this article and are available from the corresponding author on reasonable request.

## Abbreviations

AAM: Alternatively-activated Macrophages, CAM: Classically-Activated Macrophages, BMDM: Bone Marrow-Derived Macrophages, 1X PBSTw: 0.1% Tween-20 PBS, IL-4: Interleukin-4, IFN-γ, Interferon- γ, 1X PBSTr: 0.1% Triton X-100 PBS.
